# Recovery pulse rate and cardiovascular function indices in young female adults following orthostasis

**DOI:** 10.14440/jbm.2024.0127

**Published:** 2025-08-01

**Authors:** Mayowa Jeremiah Adeniyi, Ayoola Awosika

**Affiliations:** 1 Departments of Physiology, Faculty of Basic Medical Sciences, Federal University of Health Sciences, Otukpo, Benue 972261, Nigeria; 2 Department of Family Medicine, College of Medicine, University of Illinois Peoria, Bloomington, Illinois 61606, United States of America

**Keywords:** Recovery pulse rate, Orthostasis, Autonomic response, Blood pressure, Reclining sitting position

## Abstract

**Background:**

Recovery pulse rate (RPR) and other cardiovascular indices – such as heart rate variability and blood pressure recovery – are underutilized tools in assessing autonomic and cardiovascular adaptability to orthostasis. While orthostatic hypotension is well-documented, the prognostic significance of delayed heart rate recovery and impaired autonomic compensation remains insufficiently explored. Emerging evidence suggests that abnormal RPR may predict cardiovascular morbidity and autonomic dysfunction; however, standardized clinical guidelines for its interpretation are lacking. Bridging this gap could enhance early detection of dysautonomia and cardiovascular risk stratification.

**Objective:**

This study aimed to examine the pattern of RPR and cardiovascular function indices in healthy young female adults following 10 min of upright standing.

**Methods:**

This study evaluated post-orthostatic cardiovascular indices, including RPR measured at two intervals: 10 – 20 s and 21 – 31 s after returning to a reclining sitting position. A total of 35 healthy females were selected for the study, and appropriate inclusion was duly considered. Blood pressure, pulse rate, and other parameters were measured at baseline, after 10 min of standing, and after returning to a reclining sitting position using standard procedures. The first and second RPRs were calculated as the difference between the orthostatic pulse rate and the pulse rate measured during the two intervals, respectively, after returning to a reclining sitting position.

**Results:**

There was no significant difference between the first and second RPRs. Among the cardiovascular parameters, only systolic blood pressure and pulse pressure measured after the second RPR were significantly higher than baseline values. In addition, neither the first nor the second RPR correlated with body weight, height, or body mass index.

**Conclusion:**

No significant difference was found in autonomic response during the 10 – 20 s and 21 – 31 s post-orthostatic periods in young adult females. Incorporating RPR and related indices into clinical practice provides a non-invasive, cost-effective method to identify and monitor autonomic and cardiovascular dysfunction. This can guide therapeutic strategies, such as fluid management, exercise rehabilitation, or pharmacological interventions, tailored to improve autonomic balance and cardiovascular resilience.

## 1. Introduction

During physical activity, sympathetic autonomic dominance leads to increases in heart rate, blood pressure, and other indices of cardiovascular and respiratory function to meet the increased demand for oxygen, glucose, and other essential biochemicals, as well as to facilitate the elimination of carbon dioxide, heat, and other metabolic waste products.^1^-^5^ Cardiovascular recovery typically refers to the speed at which these physiological parameters return to resting values following the cessation of physical activity.^6^ Due to its temporal specificity, cardiovascular recovery acts as a non-invasive predictor of future or ongoing morbidity, even in asymptomatic individuals.^7^

For instance, delayed heart rate recovery (<12 beats per minute [bpm]) 1 min post-exercise has been associated with cardiovascular morbidity and mortality. A prospective cross-sectional study involving 208 patients conducted by Ghaffari *et al*.^8^ revealed a relationship between abnormal heart rate recovery 1 min post-exercise and the severity of major epicardial coronary events. Nocturnal blood pressure and 1-min post-exercise heart rate recovery were reported to be slower in white coat hypertensive non-dipper patients compared to their counterparts with white coat hypertension, whose blood pressure fell rhythmically at night.^9^,^10^ Okutucu *et al*.^11^ found that the mean 1-min heart rate recovery values were higher in a group of normotensive individuals with nighttime declines in blood pressure than in a normotensive non-dipper group.

Other than vigorous physical activity, many factors affect heart rate recovery. In a study involving 65 arsenic-exposed individuals and 35 non-exposed healthy controls, a lower 1-min post-exercise heart rate recovery was observed in the arsenic-exposed group compared to the healthy, unexposed controls.^12^ The timing of the recovery test is also critical and should not be underestimated. Savonen *et al*.^13^ found that delayed 2-min post-exercise heart rate recovery following a cycle ergometer test independently predicted mortality in healthy middle-aged men, even after adjusting for demographic and clinical factors. In a United Kingdom study involving Biobank participants, decreased post-exercise heart rate recovery after 10 s was identified as a predictor of clinical outcomes superior to heart rate recovery measured at later time intervals.^14^

Like exercise, orthostasis elicits a sympathetic response.^15^-^17^ Upon cessation of standing (*i*.*e*., postural transition to sitting or lying), sympathetic signals are withdrawn, and parasympathetic tone is activated.^18^-^22^ Shirsath *et al*.^23^ reported that slower recovery of diastolic and systolic blood pressure (SBP) in the early phase after standing was associated with accelerated brain aging in older individuals. A study by James *et al*.^24^ demonstrated a positive correlation between mean heart rate changes measured every 10 s for 1 min following both exercise and orthostatic stress. A longitudinal cohort study conducted by McCrory *et al*.^25^ in older individuals (≥50 years old) found that heart rate recovery in the first 20 s after 3 min of standing was a strong predictor of mortality. Specifically, a slower heart rate recovery between 10 and 20 s post-standing increased the hazard of mortality by 6%. However, concerns regarding the generalizability and applicability of these findings persist, as the study involved only participants aged 50 years and above and limited the orthostatic exposure to 30 min. Therefore, it is important to divert attention toward evaluating the rapidity of heart rate recovery in younger females following protracted orthostasis.

In stress testing, abnormal recovery pulse rates (RPRs) may indicate subclinical cardiac conditions or autonomic insufficiency. Abnormal RPRs or prolonged recovery times may signal underlying autonomic dysfunction or cardiovascular deconditioning, rendering this a valuable clinical tool for assessing disorders, such as postural orthostatic tachycardia syndrome, orthostatic hypotension, and heart failure. Other indices – including heart rate variability (HRV), baroreflex sensitivity, and blood pressure responses – complement RPR in assessing cardiovascular adaptability. For instance, a reduced HRV or impaired baroreflex sensitivity during orthostasis suggests autonomic neuropathy, commonly observed in diabetes mellitus or neurodegenerative conditions, such as Parkinson’s disease. Clinically, such findings may precede symptomatic orthostatic hypotension, thereby enabling early intervention.

While orthostatic cardiovascular responses are well-studied, the role of RPR and other cardiovascular parameters in young female adults remains unclear. Data are limited on how these parameters respond after 10 min of orthostasis and their potential implications for autonomic function and cardiovascular risk. Since females are known to exhibit lower orthostatic tolerance than males, this study aimed to examine the pattern of RPR and cardiovascular function indices in healthy young female adults following 10 min of upright standing.

## 2. Materials and methods

### 2.1. Study design

The experimental study was conducted in the Technologically Enhanced Laboratory of the Department of Physiology, College of Medical Sciences, Federal University of Health Sciences, Otukpo, Nigeria. A total of 55 young adult females were recruited using respondent-driven sampling due to the rigor of the study protocol. Among them, 35 apparently healthy participants – with an average age of 19.5 years – satisfied the inclusion criteria and were selected for the study.

### 2.2. Inclusion and exclusion criteria

Written informed consent was obtained from each participant before their enrollment into the study. A well-structured questionnaire was administered, followed by a physical examination. Participants were included based on the following criteria: Female sex, age between 16 and 21 years, SBP between 90 and 119 mmHg, diastolic blood pressure (DBP) between 60 and 80 mmHg, pulse rate of 60 – 100 bpm, and respiratory rate of 12 – 20 cycles/min. The included participants also met the criterion of moderate physical activity (defined as 150 min/week of moderate-intensity aerobic exercise) and all were euhydrated (*i*.*e*., optimally hydrated).

Excluded participants included those with a medical history of respiratory, cardiovascular, autonomic, renal, hepatic, or metabolic diseases, as well as any anatomical deformities. In addition, individuals with a history of habitual smoking, alcohol or caffeine consumption, or medication use were eliminated. Participants with musculoskeletal abnormalities, elevated blood pressure, or male sex were also removed from the study.

### 2.3. Experimental protocol

The study was conducted at room temperature between 8:00 a.m. and 10:00 a.m. following an overnight fasting. The participants were trained on how to perform orthostatic tasks. They were also briefed on how to report their feelings. Before the commencement of the experiment, all participants were asked to relax for 10 min. Resting blood pressure, pulse rate, and anthropometric data – including weight, height, and BMI – were recorded and expressed as mean ± standard error of the mean (SEM).

#### 2.3.1. 10-min orthostasis

Each participant transitioned to upright posture from a reclining sitting position and remained standing without swaying for a duration of 10 min. A stopwatch was started as the participants stood up and stopped after they had maintained the standing position for 10 min. Orthostatic pulse rate, orthostatic blood pressure, and orthostatic peripheral oxygen saturation were measured while in the standing position.

#### 2.3.2. RPR determination

Using a stopwatch and the palpatory technique, the first pulse rate was measured during the 10 – 20 s interval after returning to a reclining sitting position (*i*.*e*., the 10 – 20 s time interval). The first RPR was then calculated by subtracting the pulse rate recorded during the 10 – 20 s time interval from the orthostatic pulse rate.

Subsequently, the second pulse rate was measured during the 21 – 31 s time interval after returning to a reclining sitting position. The second RPR was determined by subtracting the pulse rate recorded during the 21 – 31 s time interval from the orthostatic pulse rate.

Immediately after the second pulse rate measurement in the reclining sitting position, blood pressure and peripheral oxygen saturation were recorded as post-orthostatic readings.

#### 2.3.3. Measurement of pulse rate and blood pressure

Pulse rate was determined at the radial artery using the palpatory technique. Baseline readings were recorded in the reclining sitting position.

Blood pressure was measured on the arm, approximately one inch above the elbow, using an automated Omron BP7000 Evolve Wireless Upper Arm Sphygmomanometer (Iris Global Care, China). Baseline readings were recorded in the reclining sitting position, as previously reported.^26^,^27^ Additional blood pressure measurements were taken after 10 min of orthostasis (in the standing position) and immediately following the second pulse rate recording.

Pulse pressure was calculated by subtracting the DBP from the systolic pressure. Mean arterial blood pressure was calculated as DBP plus one-third of the pulse pressure.

The shock index was calculated by dividing the pulse rate by SBP. The double product was obtained by multiplying the mean arterial blood pressure by the pulse rate.

#### 2.3.4. Determination of peripheral oxygen saturation

Peripheral oxygen saturation was measured by using the digital pulse oximeter MD300C25 (American Diagnostic Corporation, USA).

#### 2.3.5. Determination of anthropometric indices

Body weight was measured using a weighing scale (Hanson, China) to the nearest 0.5 kg. Height was measured using a meter ruler calibrated in inches. The BMI of each participant was calculated using the following formula:


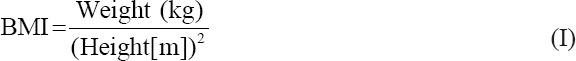


### 2.4. Statistical analysis

Statistical analysis was performed using the Statistical Package for the Social Sciences (SPSS), version 23 (IBM, United States). Statistical tests were conducted using Student’s *t*-test. A *p*-value of < 0.05 was considered statistically significant. Correlation between variables was assessed by using Pearson’s correlation coefficient.

## 3. Results

### 3.1. Anthropometrical characteristics of the participants

The weight, height, and BMI of the participants were measured and are summarized in Table 1.

**Table 1:** Anthropometric characteristics of the participants

Parameters	Mean±SEM
Weight (kg)	57.7±2.000
Height (m)	1.6±0.030
Body mass index	20.2±0.520

Abbreviation: SEM: Standard error of the mean.

### 3.2. RPR in young adult female participants after prolonged orthostasis

**Figure 1:**
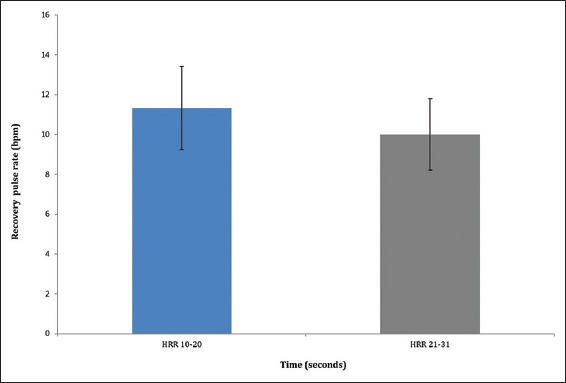
Recovery pulse rate in young adult female participants measured during the 10 – 20 and 21 – 31 s intervals following prolonged orthostasis. Abbreviations: bpm: Beats per minute; HRR: Heart rate recovery.

No significant difference was observed between RPRs measured during the 10 – 20 s and 21–31 s time intervals (*p*>0.05) (Figure 1).

### 3.3. Peripheral oxygen saturation in young adult female participants after prolonged orthostasis

**Figure 2:**
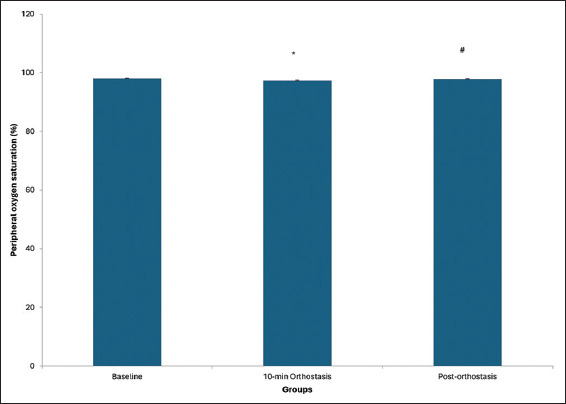
Peripheral oxygen saturation in young adult female participants following prolonged orthostasis. Asterisk (*) and hash symbol (#) represent significant differences from baseline and post-orthostatic recovery values, respectively (*p*<0.05).

The 10-min orthostasis resulted in a significant decrease in peripheral oxygen saturation compared to baseline (*p*<0.05) (Figure 2). No significant difference was found between baseline and post-orthostatic recovery values (*p*>0.05). However, oxygen saturation during 10-min orthostasis was observed to be significantly lower than that post-orthostatic recovery.

### 3.4. Cardiovascular parameters in young adult female participants after prolonged orthostasis

Compared to baseline, 10 min of orthostasis resulted in significant increases (*p*<0.05) in SBP, DBP, pulse pressure, mean arterial blood pressure, shock index, double product, and pulse rate (Table 2). SBP and pulse pressure were also significantly elevated during the post-orthostasis phase compared to the baseline phase. During the 10-min orthostasis, SBP, DBP, mean arterial blood pressure, shock index, double product, and pulse rate were all significantly higher compared to the post-orthostasis phase.

**Table 2:** Cardiovascular parameters in young adult female participants following prolonged orthostasis

Cardiovascular parameters	Baseline (mean±SEM)	10-min orthostasis (mean±SEM)	Post-orthostasis (mean±SEM)
Systolic blood pressure (mmHg)	108.6±0.967	116.3±1.096*	111.6±1.283*^#^
Diastolic blood pressure (mmHg)	70.6±0.685	76.4±0.776*	72±1.229^#^
Pulse pressure (mmHg)	38±0.298	39.9±0.378*	39.6±0.747*
Mean arterial blood pressure (mmHg)	83.2±0.778	89.7±0.877*	85.2±1.197^#^
Shock index (bpm/mmHg)	0.75±0.014	0.79±0.020*	0.74±0.007^#^
Double product (bpm×mmHg)	6769.5±128.766	8254.2±182.052*	6988.9±146.337^b^
Pulse rate (bpm)	81.3±1.300	92.0±2.000*	82.0±0.895^#^

Note: Asterisk (*) and hash symbol (#) represent significant differences from baseline and post-orthostatic recovery values, respectively (*p*<0.05). Abbreviations: bpm: Beats per minute; SEM: Standard error of the mean.

### 3.5. Correlation between RPR and anthropometric indices

Both RPRs measured during the 10 – 20 s and 21 – 31 s intervals after standing showed no significant correlation with weight, height, and BMI (*p*>0.05) (Table 3).

**Table 3:** Correlation between recovery pulse rate and anthropometric indices

Pearson’s correlation (*r*)	Anthropometric indices		

	Weight	Height	Body mass index
First recovery pulse rate (10 – 20 s post-orthosis)	−0.007	0.000	0.014
Second recovery pulse rate (21 – 31 s post-orthosis)	−0.006	−0.000	0.012

## 4. Discussion

The rate at which the indices of cardiovascular function return to resting values after physical exertion plays a crucial role in evaluating the functional status of the cardiovascular and autonomic nervous systems, as well as overall well-being.^6^,^7^ Specifically, post-exercise heart rate recovery is an important tool in both health assessment and the diagnosis and prognostication of cardiovascular conditions. Impaired heart rate recovery at 1 min post-exercise is a predictor of the severity of coronary artery disease.^8^ Delayed heart rate recovery is also associated with inflammatory markers, particularly C-reactive protein, even in apparently healthy individuals.^6^ Among patients without coronary artery disease, a slow heart rate recovery has been identified as an independent predictor of vasospastic angina.^28^ In addition, individuals with microalbuminuria exhibit reduced heart rate recovery.^7^ The aim of the present study was to investigate RPR and indices of cardiovascular function in young female adults after 10 min of standing.

Increases in SBP, DBP, pulse pressure, pulse rate, mean arterial blood pressure, and shock index, along with a reduction in peripheral oxygen saturation observed following protracted standing, have been extensively documented.^17^,^22^,^27^,^29^ These findings suggest that sympathetic activation is mediated by baroreceptor inactivation – a mechanism initiated by blood pooling in the lower extremities, diminished venous return, and reduced circulating blood volume.^27^,^30^ An inadequate compensatory response may lead to dizziness and syncope.^1^ In addition to baroreceptor involvement, prolonged standing may stimulate cortisol production from the zona fasciculata of the adrenal cortex and renin secretion from juxtaglomerular cells in the kidney.^1^ Cortisol – through its mineralocorticoid activity – promotes sodium and water retention, thereby increasing intravascular volume. Renin triggers the production of angiotensin II and aldosterone.^1^ Collectively, these hormones enhance glomerular filtration rate, sodium and water retention, and blood volume, ultimately improving cardiac output.^1^

In this study, the first RPR – measured during the 10–20 s interval after returning to a reclining sitting position – did not differ significantly from the second RPR, measured during the 21 – 31 s interval. McCrory *et al*.^25^ previously reported that heart rate recovery within the first 30 s post-exercise predicts mortality. However, their study focused on individuals over 50 years of age, and the orthostatic duration employed was 3 min. In contrast, the present study involved a 10-min orthostasis and included female participants aged 16 – 21 years. Therefore, further studies are warranted to clarify the precise definition of RPR following prolonged standing in both healthy and diseased states.

Furthermore, RPR measured during both the 10 – 20 and 21 – 31 s intervals revealed no significant correlation with body weight, height, or BMI in the study population. Although the present findings are confined to young adult females aged 16 – 21 years, they suggest that early heart rate recovery post-orthostasis may be independent of these anthropometric parameters. Nonetheless, further studies are needed to validate these findings. The present study indicates that post-orthostatic heart rate recovery occurs within the first 31 s – potentially extending the 30-s window documented in the literature.

In the study, post-orthostatic indices of cardiovascular function were measured immediately after the second RPR. Although post-orthostatic SBP and pulse pressure were significantly lower than the corresponding orthostatic values, they were significantly higher than baseline values. Lee *et al*.^31^ measured recovery blood pressure 4 min after the cessation of exercise, and this was reported as a predictor of mortality in middle-aged healthy individuals. However, there is no established consensus regarding the optimal time point at which recovery blood pressure should be measured following physical exertion. In contrast, DBP, mean arterial blood pressure, shock index, and peripheral oxygen saturation measured immediately after the second RPR did not differ significantly from baseline values.

While this study offers valuable insight into the clinical relevance of RPR and orthostatic response, several limitations remain. The inclusion of a small sample size consisting solely of healthy participants may limit the generalizability of the findings, potentially overlooking individuals at greater risk for impaired orthostatic regulation. In addition, female participants were specifically recruited due to their typically lower orthostatic tolerance. Therefore, future research should incorporate a larger, more diverse population across genders and varying health conditions to enhance the applicability and validity of the findings.

## 5. Conclusion

RPR and other cardiovascular indices in response to orthostasis provide valuable insights into the adaptive capacity of the cardiovascular and autonomic systems. These metrics are clinically significant for diagnosing and monitoring conditions, such as autonomic dysfunction, postural orthostatic tachycardia syndrome, orthostatic hypotension, and heart failure. Impaired responses may reveal early signs of autonomic neuropathy, hypovolemia, or neurodegenerative disorders, thereby guiding timely intervention and management.

In this study, there was no significant difference in autonomic response during both the 10 – 20 s and 21 – 31 s intervals following orthostasis in young adult females. Age-related physiological differences may have influenced these indices, as younger individuals typically exhibit more robust autonomic responses compared to older adults, who often demonstrate reduced cardiovascular adaptability due to age-associated declines in baroreceptor sensitivity and vascular compliance. Therefore, integrating these indices into clinical practice may support early diagnosis, individualized management, and targeted interventions tailored not only to specific conditions but also to the patient’s age and overall physiological status. However, further research is needed to clarify the precise characteristics of RPR following prolonged standing in both healthy and diseased states.

## Data Availability

The data generated in the present study are included in the figures and/or tables of this article.
